# Supplementary material for: A deep learning method for replicate-based analysis of chromosome conformation contacts using Siamese neural networks
Source: Nat Commun. 2023 Aug 17;14:5007. doi: 10.1038/s41467-023-40547-9 (PMC10435498; doi:10.1038/s41467-023-40547-9)
Supplement: Supplementary file 1 — Supplementary info [file 41467_2023_40547_MOESM1_ESM.pdf]

Supplementary Materials and Figures

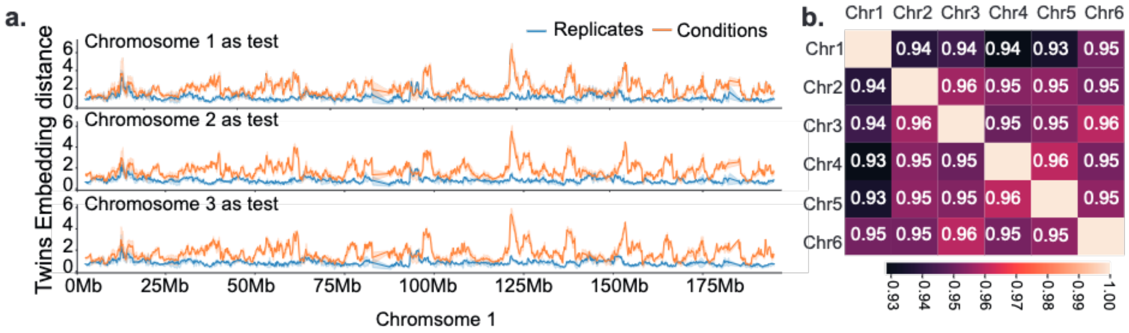

Figure S1. The choice of test chromosome does not impact the Twins embedding distance.
a. Twins embedding distances for conditions (orange) and replicates (blue) are plotted for thymocytes at CD4 SP versus DP stages of differentiation are depicted across chromosome 1 for networks trained with a different choice of test chromosome. Data are presented as mean values ± the 95% confidence interval.
b. The correlation coefficient for the genome wide embedding distances calculated using networks each with a different choice of test chromosome from chromosomes 1-6.

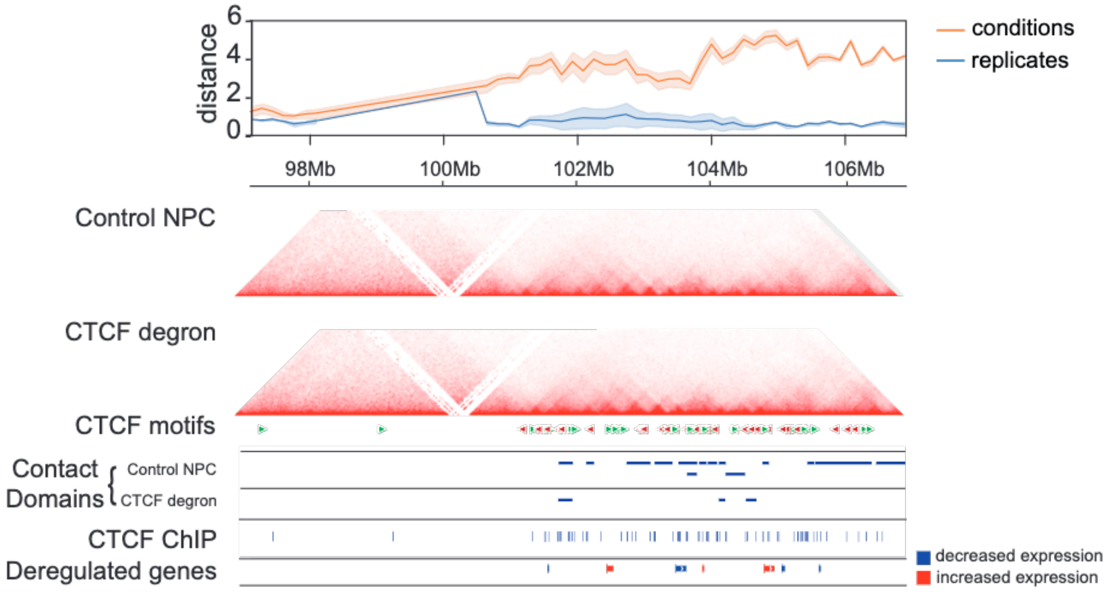

Figure S2. Twins embedding distances compared with the locations of deregulated genes CTCF motifs contact domains and CTCF ChIP.
Illustration that regions with high Twins scores after CTCF depletion in neuronal progenitor cells are characterised by changes in contact domains, deregulated genes, and a high density of CTCF motifs in addition to a high density of CTCF binding detected by ChIP-seq. Twins embedding distances are presented as mean values ± the 95% confidence interval.

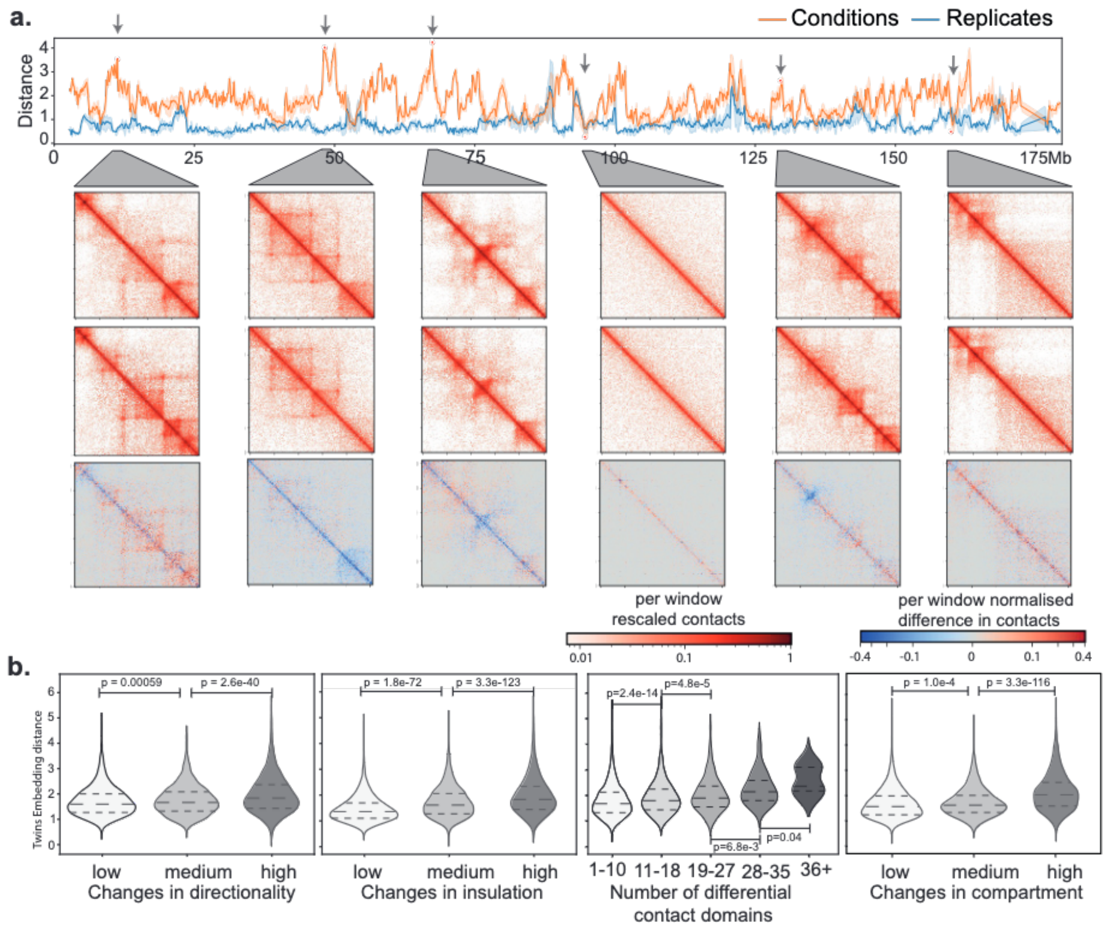

Figure S3. Twins embedding distance finds meaningful changes in T-cell differentiation.

a. Hi-C maps of 4 representative genomic regions with high Twins scores and 2 representative genomic regions with low Twins scores. Twins embedding distances are presented as mean values ± the 95% confidence interval.

b. Twins scores reflect changes in Hi-C features during T cell development. Comparison of Twins embedding distance to changes in directionality, insulation, differential contact domains and changes in compartment identity. Mean changes in directionality at domain boundaries, total insulation and changes in compartment are quantified for each window and placed into three equal sized bins (low, medium and high) by percentile. Number of differential contact domains are counted for each window and placed into bins ([1-10], [11-18], [19-27], [28-35] and 36+) each bin has a variable number of regions (5786, 2475, 672, 113, 19). All p-values are assigned using a two-sided t-test.

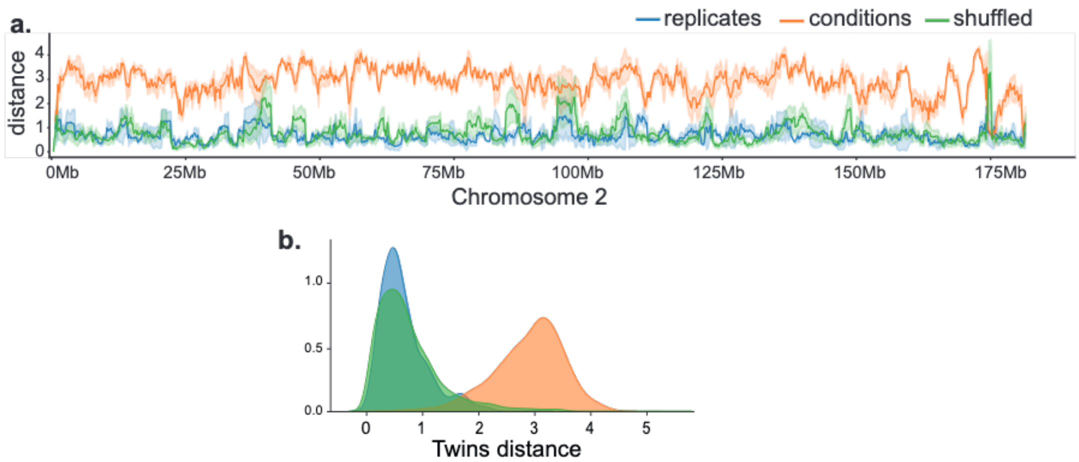

Figure S4. A trained Twins network recognises erroneous inputs
a. Twins embedding distance for liver network across Chromosome 2 for NIPBL ko/TAM replicates, NIPBL ko vs TAM condition pairs and a comparison of two shuffled Hi-C maps containing half the reads from TAM mixed with half the reads from the NIPBL ko. Data are presented as mean values ± the 95% confidence interval.
b. Overall distance distributions for the shuffled, replicate and condition pairs in the NIPBL knock out vs the tamoxifen control.

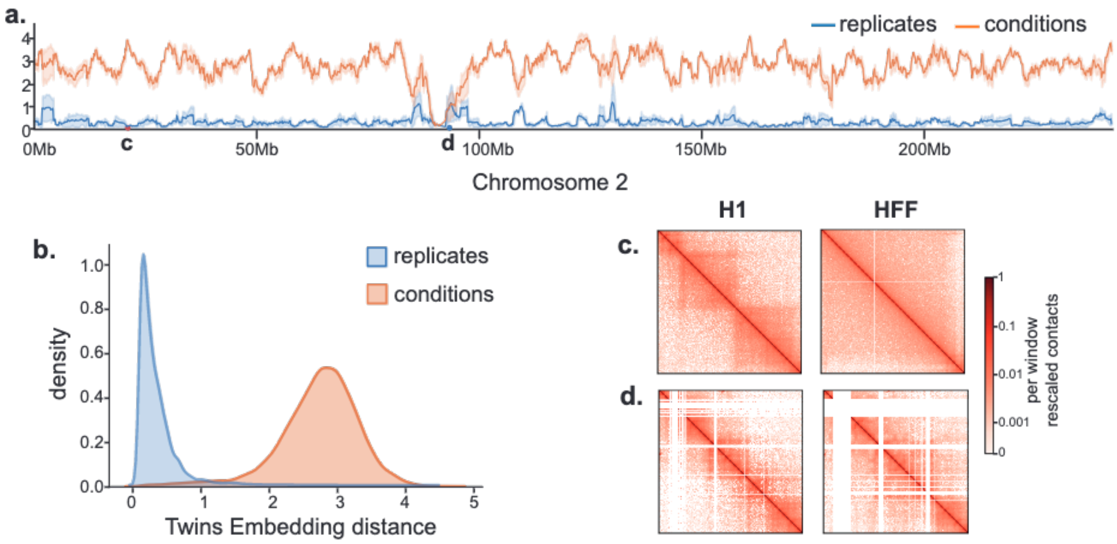

Figure S5. Increased data resolution enhances Twins performance.
a. Twins embedding distance along chromosome 2 for network trained on Micro-C with the locations of the regions displayed in c and d marked. Data are presented as mean values ± the 95% confidence interval.
b. Twins embedding distance distribution genome wide for replicate and condition pairs from Micro-C data.
c. Example of a region with a high level of conformational change according to the Twins embedding distance.
d. Example of a region a low level of conformational change according to the Twins embedding distance.

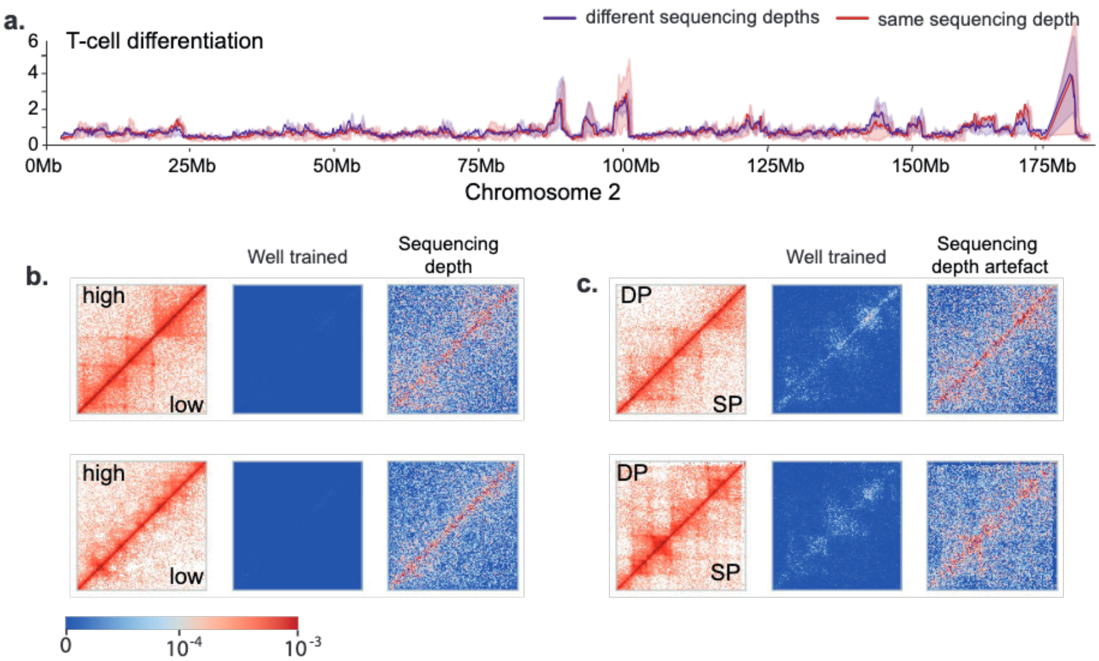

Figure S6. Well trained networks are robust to sequencing depth artefacts.
a. Twins embedding distances across chromosome 2 for T-cell differentiation network for comparison between high-depth and low-depth Hi-C windows. Data are presented as mean values ± the 95% confidence interval.
b. Example integrated gradient maps for the well trained and sequencing depth artefact networks on high-depth and low-depth Hi-C at chr2:48480000-51040000 and chr2:163040000-165600000 respectively.
c. Example integrated gradient maps for the well trained and sequencing depth artefact networks on DP and CD4 SP thymocyte Hi-C at chr2:48480000-51040000 and chr2:172320000-174880000 respectively.

Resolution R	Window Size S	Stride	Stride Length
2kb	512kb	4	128kb
5kb	1.28Mb	8	160kb
10kb	2.56Mb	16	160kb
25kb	6.4Mb	32	200kb

Table S1. Parameters for window stride and size for each resolution.

Stride density per window	stride size (kb)	Average train time	File size (per file)
2	1280	1m47s	397MB
4	640	3m26s	796MB
8	320	6m48s	1.55GB
16	160	12m42s	3.10GB
32	80	28m21s	6.22GB
64	40	$\sim$ 1hr	12.44GB
128	20	$\sim$ 2hrs	24.88GB

Table S2. The effect of varying the stride for windows of size 2.56Mb and 10kb resolution a mouse dataset with average network train times across 5 random seeds. s

T-cell differentiation	R1	R2
DP	244438962	223193977
SP	222906061	209206741
DP high depth	417830413	427257794

Nipbl deletion	R1	R2
TAM	51998355	75626897
WT	76277577	65903921
NCAP2	67503333	63246470
NIPBL	65081892	72757890

CTCF degron	R1	R2
Control	845858980	838742819
Auxin	737448939	843972188

Micro-C	R1	R2
midrule H1	1470519840	1749983591
HFF	3049701785	1508233177

Table S3. Sequencing depths for each dataset utilised in the study.
